# Prognostic and immunological role of adaptor related protein complex 3 subunit mu2 in colon cancer

**DOI:** 10.1038/s41598-023-50452-2

**Published:** 2024-01-04

**Authors:** Qianqian Jin, Jiahao Feng, Yang Yan, Yong Kuang

**Affiliations:** 1 Department of Cardiovascular Surgery, The First Affiliated Hospital of Xi’an Jiaotong University, 277 West Yanta Road, Xi’an, 710061 Shaanxi P. R. China; 2 Research Centre, The Seventh Affiliated Hospital of Sun Yat-Sen University, Shenzhen, 518107 China; 3 Digestive Disease Center, The Seventh Affiliated Hospital of Sun Yat-Sen University, Shenzhen, 518107 China

**Keywords:** Cancer microenvironment, Gastrointestinal cancer, Tumour immunology

## Abstract

The expression levels and prognostic role of AP3M2 in colorectal adenocarcinoma (CRAC) have yet to be fully unveiled. Our study comprehensively investigated the clinical significance of AP3M2 in colorectal cancer through an extensive bioinformatics data mining process (TCGA, GEO, GEPIA, Timer, Ualcan, ROCPLOT, and David), followed by experimental validation. We found AP3M2 is a cancer gene, which can be used to distinguish between colorectal cancer and colorectal adenomas, liver metastasis, lung metastasis, colorectal polyp. Higher AP3M2 expression levels were associated with longer overall survival in colon adenocarcinoma. AP3M2 might be the primary biomarker for oxaliplatin in colon cancer and an acquired resistance biomarker for oxaliplatin and 5-fu. AP3M2 was positively associated with CD274, CTLA4. AP3M2 might be associated with T-cell, NF-kappaB transcription factor activity, and response to hypoxia. AP3M2 could predict chemotherapy effectiveness and prognosis for colon cancer patients. AP3M2 might inhibit tumor growth via influencing tumor-infiltrating immune cells in the context of Tumor microenvironment. AP3M2 plays as an oncogene in CRAC and is suggested as a new potential biotarget for therapy.

## Introduction

Colorectal cancer is a prevalent malignant neoplasm of the digestive system. With the increase in life expectancy and changes in drinking habits among Chinese individuals, both the incidence and mortality rates of colorectal cancer have been steadily rising^1^. Early detection and identification of novel biomarkers are crucial for effective management. Recently, AP3M2, previously associated with epilepsy and alcoholism^2–4^, has emerged as a potential prognostic marker for colorectal cancer^5^, It encodes a subunit of heterotetrameric adapter-associated protein complex 3 (AP-3), belonging to the medium subunit family of adaptor complexes involved in protein transport^6^. However, limited research exists on its specific relevance to colorectal cancer progression.

In this study, we explored the role of AP3M2 in colorectal cancer by using an extensive bioinformatics data mining process to determine the expression of AP3M2 in various cancers. We found the performance of AP3M2 in colon cancer (CC) and rectal cancer (RC) are quite different, to explore the role of AP3M2 gene in the survival, function and structure of CRAC, we used an extensive bioinformatics data mining process by several databases to analyze the clinical role of AP3M2 respectively.

## Results

### The expression of AP3M2 in colorectal adenocarcinoma (CRAC)

**Figure 1:**
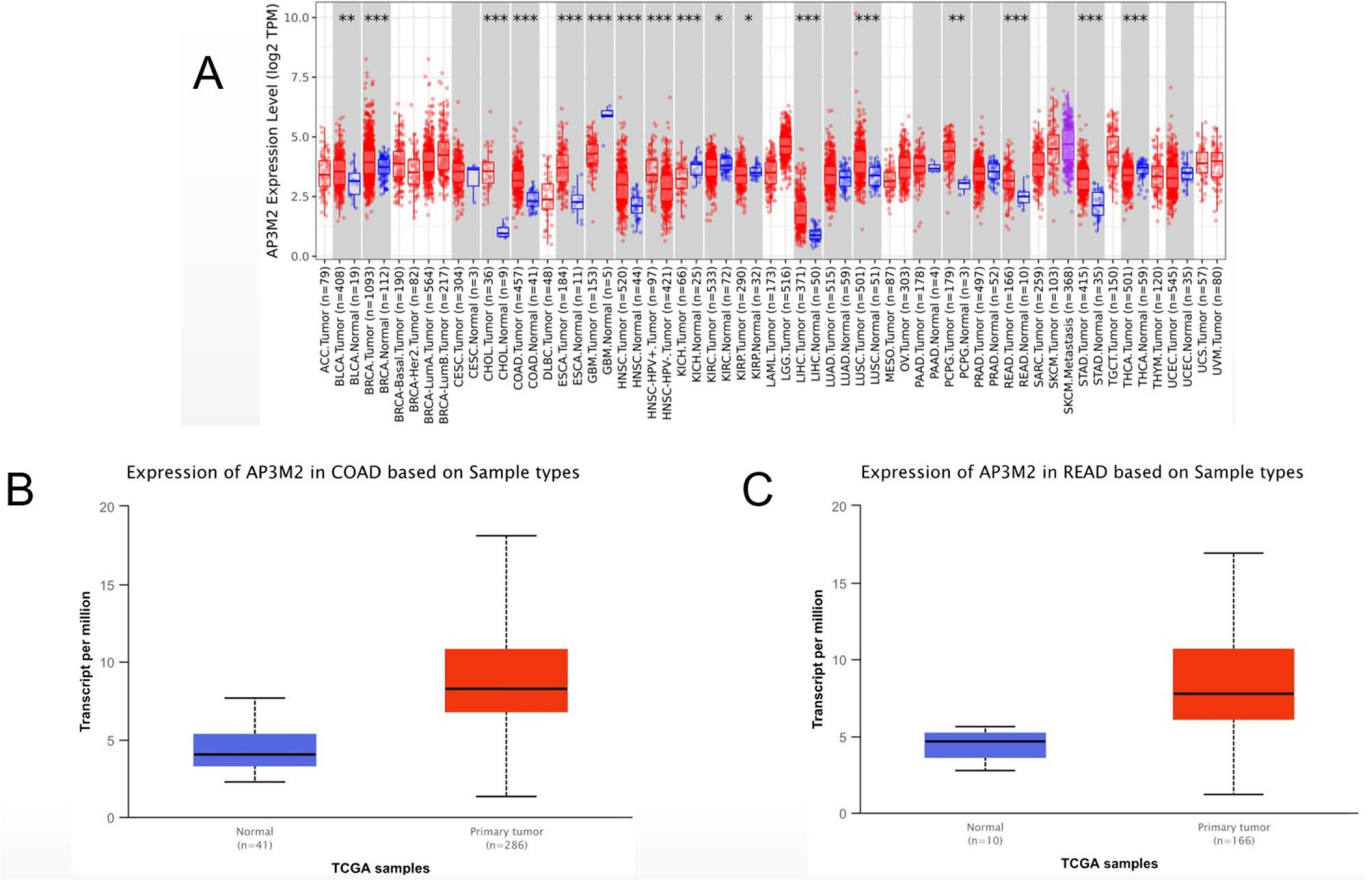
The relationship between AP3M2 expression and Tumor types: (**a**) The transcription level of AP3M2 in various cancer types (TIMER database) (*p*-value < 0.01). (**b**) The box plot comparing specific AP3M2 expression in normal (left plot) and colon cancer tissues (right plot) was derived from the ualcan database. (**c**) (*p*-value < 1E−12). The box plot comparing specific AP3M2 expression in normal (left plot) and rectal cancer tissue (right plot) was derived from ualcan database. (*p*-value < 7.51E−05).

**Figure 2:**
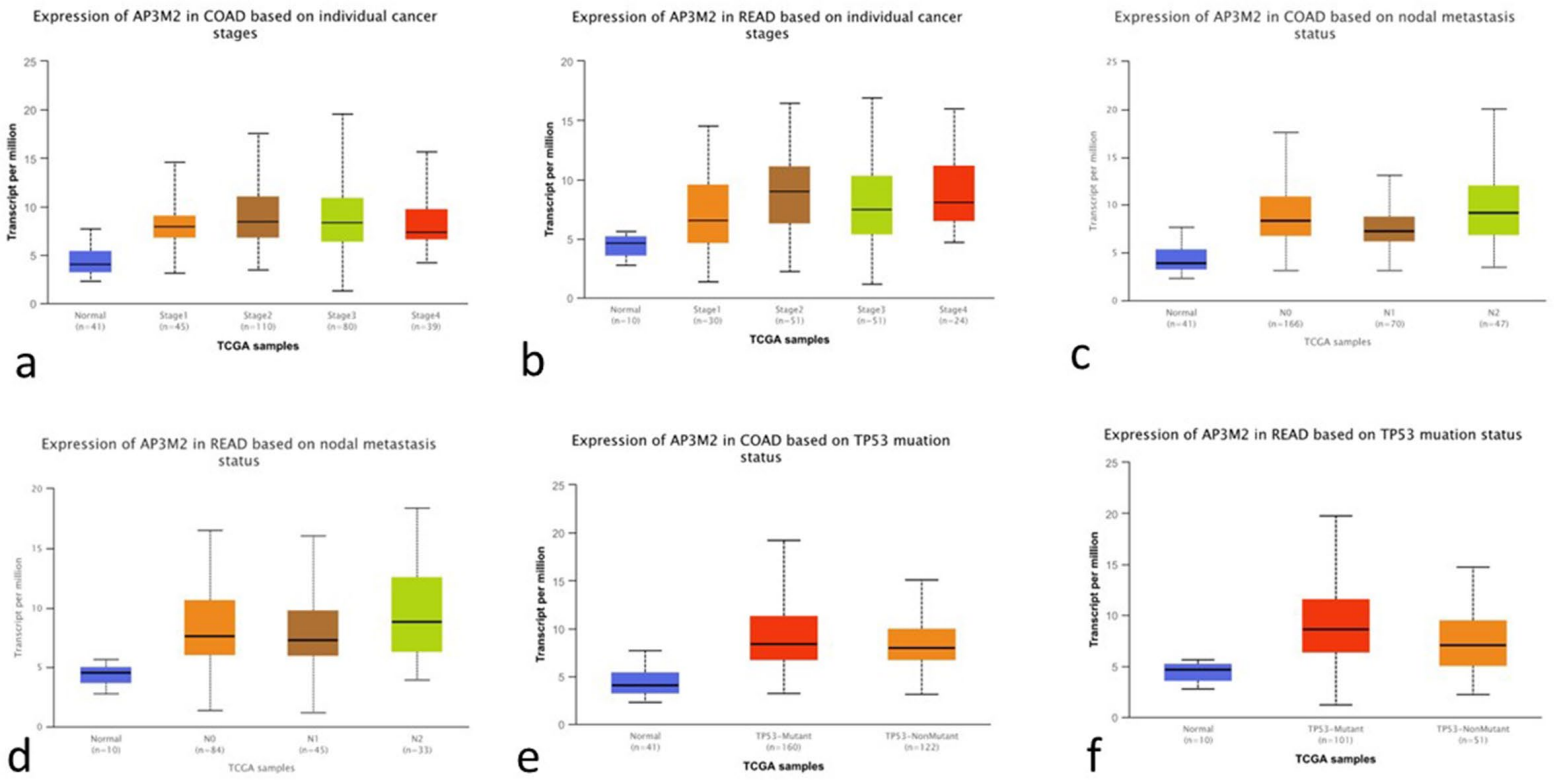
The relationship between AP3M2 expression and Clinical features: (**a**) The box plot comparing specific AP3M2 expression in different stages was derived from ualcan database in colon cancer. (**b**) The box plot comparing specific AP3M2 expression in different stages was derived from ualcan database in rectal cancer. (**c**)The box plot comparing specific AP3M2 expression in various pathologic Lymph node stages was derived from ualcan database in colon cancer. (**d**) The box plot comparing specific AP3M2 expression in various pathologic Lymph node stage was derived from ualcan database in rectal cancer. (**e**)The box plot comparing specific AP3M2 expression in different TP53 mutation status was derived from ualcan database in colon cancer. (**f**) The box plot comparing specific AP3M2 expression in different TP53 mutation status was derived from ualcan database in rectal cancer.

According to TCGA data from the TIMER database, the transcription level of AP3M2 across various cancer types showed statistical significance (*p*-value < 0.01). In colon cancer tissue, the expression level of AP3M2 was notably higher than in normal colon tissue (median 4.03 vs. 8.25 TPM—transcripts per million, *p*-value < 1E−12). Similarly, in adjacent tissue to carcinoma in rectal cancer, we observed comparable results (median 4.66 vs. 7.78 TPM, *p*-value < 7.51E−05). (Fig. 1). According to GSE database, the AP3M2 expression in colon cancer have no statistical significance (*p*-value < 0.05, |logFC|> 1) in trial results of colonic adenomas, liver metastasis, lung metastasis, colonic polyp. (colonic adenomas: GSE4183, *p*-value = 2.46E−01; GSE41258, *p*-value = 0.05; liver metastasis: GSE41258, *p*-value = 5.39E−01; lung metastasis: GSE41258, *p*-value = 8.98E−01; colonic polyp: GSE41258, *p*-value = 5.06E−05, logFC = 0.24; GSE68468, *p*-value = 1.36E−05, logFC = 3.19E−01). The expression levels of AP3M2 in colon cancer patients of different groups are similar to the expression level of rectal cancer, but they have no significantly relevant with age and gender (Supplemental Fig. 2). The expression level of AP3M2 in stage 1 to 4 colon cancer tissues was higher than that in adjacent normal tissue (stage 1 vs. stage 2 vs. stage 3 vs. stage 4: 7.95 vs. 8.51 vs. 8.42 vs. 7.40). The expression level of AP3M2 was uncorrelated between each stage. Which was also observed in rectal cancer. There were statistical significances in the AP3M2 expression levels between pathologic N0 (median 8.33), N1(median 7.28), N2 (median 9.19), and normal (median 3.94) in colon cancer. The same as rectal cancer (N0 (median 7.59), N1(median 7.27), N2 (median 8.82), and normal (median 4.58)). In addition, there were statistical significances in the AP3M2 expression levels between N1 and N2 in colon cancer (*p*-value < 0.05). The expression level of AP3M2 in different TP53 mutation status was higher than that in adjacent normal tissues (CC: normal vs. TP53 mutant vs. TP53 non-mutant: 4.03 vs. 8.45 vs. 7.97; RC: normal vs. TP53 mutant vs. TP53 non-mutant: 4.66 vs. 8.58 vs. 7.04). Nevertheless, it is correlated between the expression level of AP3M2 in different TP53 mutation status in rectal cancer while it is irrelevant in colon cancer (*p*-value < 0.05). (Fig. 2).

### The relationship between the transcription level of AP3M2 and prognosis of colorectal adenocarcinoma (CRAC)

**Figure 3:**
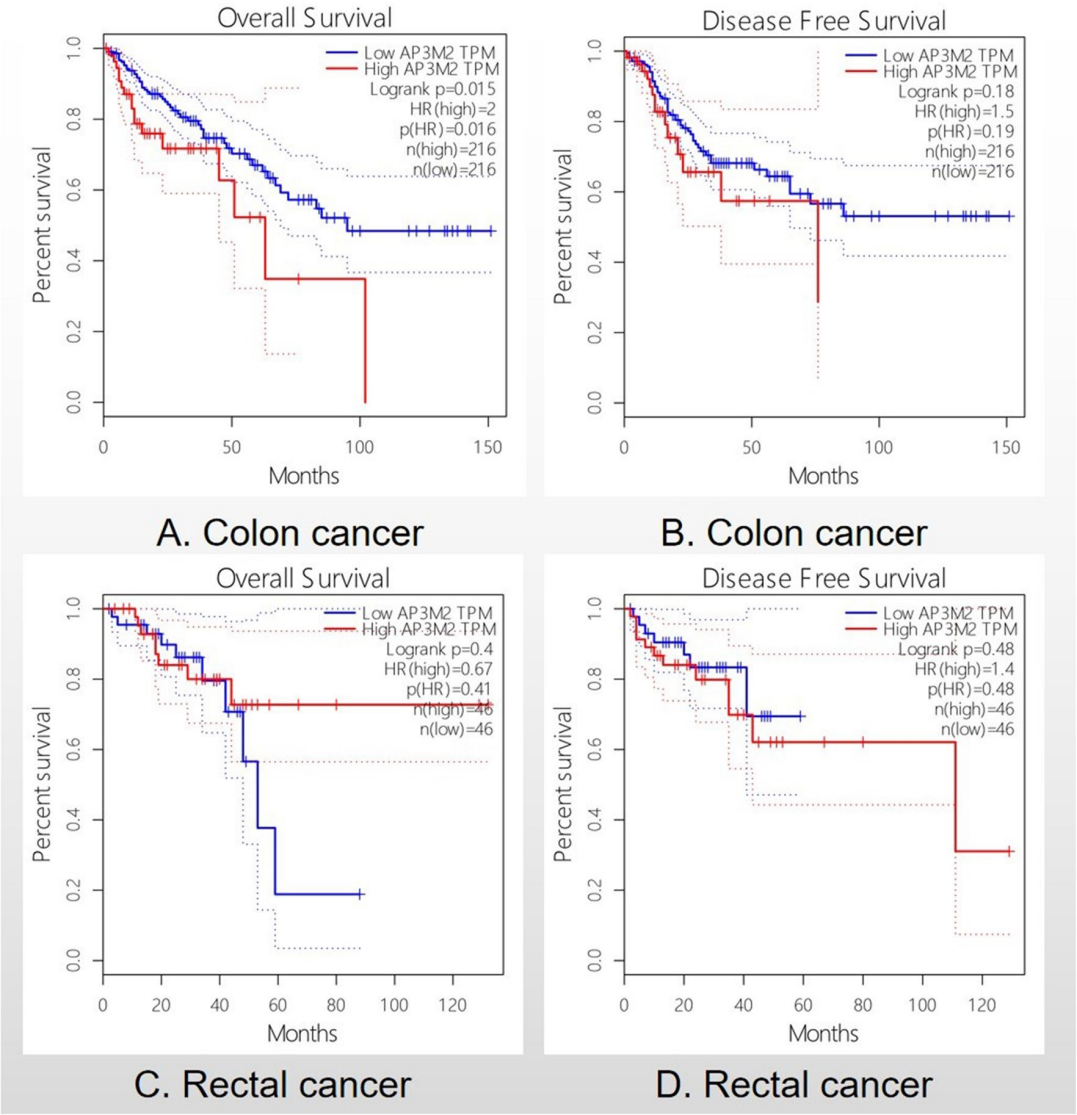
The relationship between AP3M2 expression and survival: (**a**) The relationship between AP3M2 expression and OS in colon cancer. (**b**) The relationship between AP3M2 expression and DFS in colon cancer. (**c**) The relationship between AP3M2 expression and OS in rectal cancer. (**d**) The relationship between AP3M2 expression and DFS in rectal cancer.

The relationship between the expression level of AP3M2 and patient prognosis was investigated in Colonic Neoplasm. It was observed that a higher AP3M2 expression level was significantly associated with unfavorable Overall Survival (OS) in colon cancer (*p*-value = 0.016, HR: 2), indicating a two-fold higher risk. However, no statistically significant difference was found in rectal adenocarcinoma (Fig. 3). Moreover, overexpression of AP3M2 was correlated with OS but showed no significant correlation with Disease-Free Survival (DFS). These findings suggest that elevated AP3M2 expression may serve as an indicator of poor OS specifically in colon cancer cases.

### The expression of AP3M2 between acquired drug-resistant and parental cell lines

#### The predictive values of AP3M2 in CRAC

**Figure 4:**
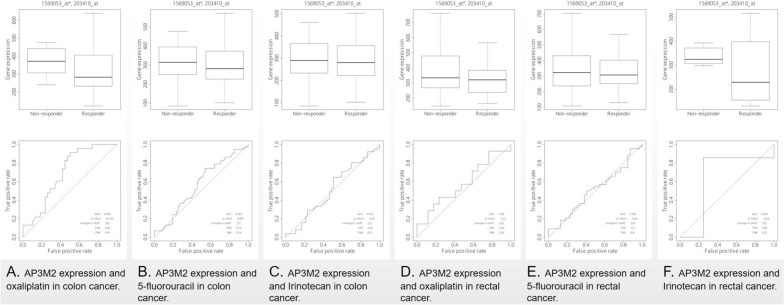
The relationship between AP3M2 expression and drug response: (**a**) The relationship between AP3M2 expression and oxaliplatin in colon cancer. (**b**) The relationship between AP3M2 expression and 5-fluorouracil in colon cancer. (**c**) The relationship between AP3M2 expression and Irinotecan in colon cancer. (**d**) The relationship between AP3M2 expression and oxaliplatin in rectal cancer. (**e**) The relationship between AP3M2 expression and 5-fluorouracil in rectal cancer. (**f**) The relationship between AP3M2 expression and Irinotecan in rectal cancer.

Based on the rocplot.plot analysis, in colon cancer patients, the expression level of AP3M2 was found to be higher in non-responders compared to responders who received oxaliplatin (AUC = 0.693, *p*-value = 2e−03). However, AP3M2 may not serve as a predictive marker for the effectiveness of 5-fluorouracil (AUC = 0.565, *p* = 0.081) and Irinotecan (AUC = 0.512, *p*-value = 0.42). Notably, a high level of AP3M2 might be associated with poor treatment response among colon cancer patients receiving oxaliplatin therapy. Conversely, no significant association between AP3M2 expression and drug response was observed among rectal cancer patients treated with oxaliplatin (AUC = 0.580, *p*-value = 0.230), 5-fluorouracil (AUC = 0.509, *p*-value = 0.440), or Irinotecan (AUC = 0.643, *p*-value = 280) (Fig. 4).

#### The expression of AP3M2 between acquired drug-resistant and parental cell lines

The acquisition of resistance biomarkers for trifluridine (GSE96787, logFC = 0.08, *p*-value = 0.868), oxaliplatin (GSE10405, logFC = 0.13, *p*-value = 0.36; GSE42387: HCT116: logFC = 0.10, *p*-value = 0.24; LoVo: logFC = 0.39, *p*-value = 5.39e02), BRAF inhibitor (GSE10405: HT29: logFC = 0.52, *p*-value = 0.29), trifluoro -thymidine (GSE18137: H630: logFC = 0.77, *p*-value = 0.12) and irinotecan (GSE42387: LoVo: logFC = 0.23, *p*-value = 0.33; GSE59501: LoVo:log FC = 0.59, *p*-value = 0.32; GSE42387: HCT116:log FC = 0.l7, *p*-value = 5.43e−02;G SE4238 HT29: logFC = 0.23, *p*-value = 6.94e−02) may not be applicable to AP3M2. However, AP3M2 could serve as an acquired resistance biomarker for fluorouracil in Hct-8 cell lines(GSE81008, Hct-8: logFC = 0.40, *p*-value = 1.99e−03)and oxaliplatin in HT29 cell lines(GSE42B7, HT29: logFC = 0.52, *p*-value = 9.30e−04).

#### AP3M2 might influence the prognosis of colon cancer via immune regulation

**Figure 5:**
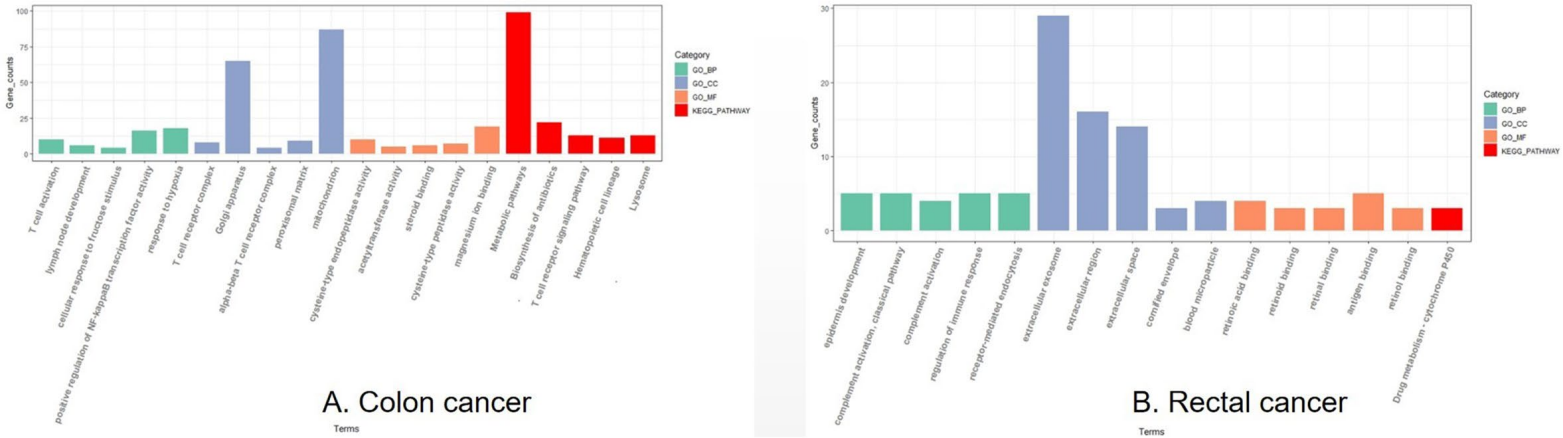
Enrichment analysis for AP3M2: (**a**) The KEGG and GO pathway in colon cancer. (**b**) The KEGG and GO pathway in rectal cancer.

According to the KEGG^7–9^ and GO enrichment analysis of genes associated with AP3M2 (r > 1 or r < − 1, *p*-value < 0.05), we identified a potential association between AP3M2 and immune regulation, particularly T-cell immunity. In terms of biological processes, AP3M2 may be involved in T cell activation, lymph node development, positive regulation of NF-kappaB transcription factor activity, and response to hypoxia. Regarding cellular components, AP3M2 may play a role in the T cell receptor complex and alpha–beta T cell receptor complex Α-β. Additionally, the KEGG pathway analysis suggests an association between AP3M2 and the T cell receptor signaling pathway (Fig. 5a).

Among the genes related to T-cell immunity (biological process: GO:0042110, T cell activation; cellular component: GO:0042101, T cell receptor complex; GO:0042105, alpha–beta T cell receptor complex; KEGG pathway: hsa04660, T cell receptor signaling pathway), seven genes (CD3E, CD3G, CD247, CD3D, CD8A, CD8B, ZAP70) were found across all these categories. Furthermore,four genes(CD3E,CD3G,CD247,CD8A) showed significant associations with AP3M2 according to Timer database results(Fig. 5b).

The results from KEGG and GO enrichment analyses regarding AP3M2(r > 1 or r < − 1, *p*-value < 0.05) in rectal cancer are presented in Fig, 6. There is no evidence suggesting an association with immune-related functions in rectal cancer.

**Figure 6:**
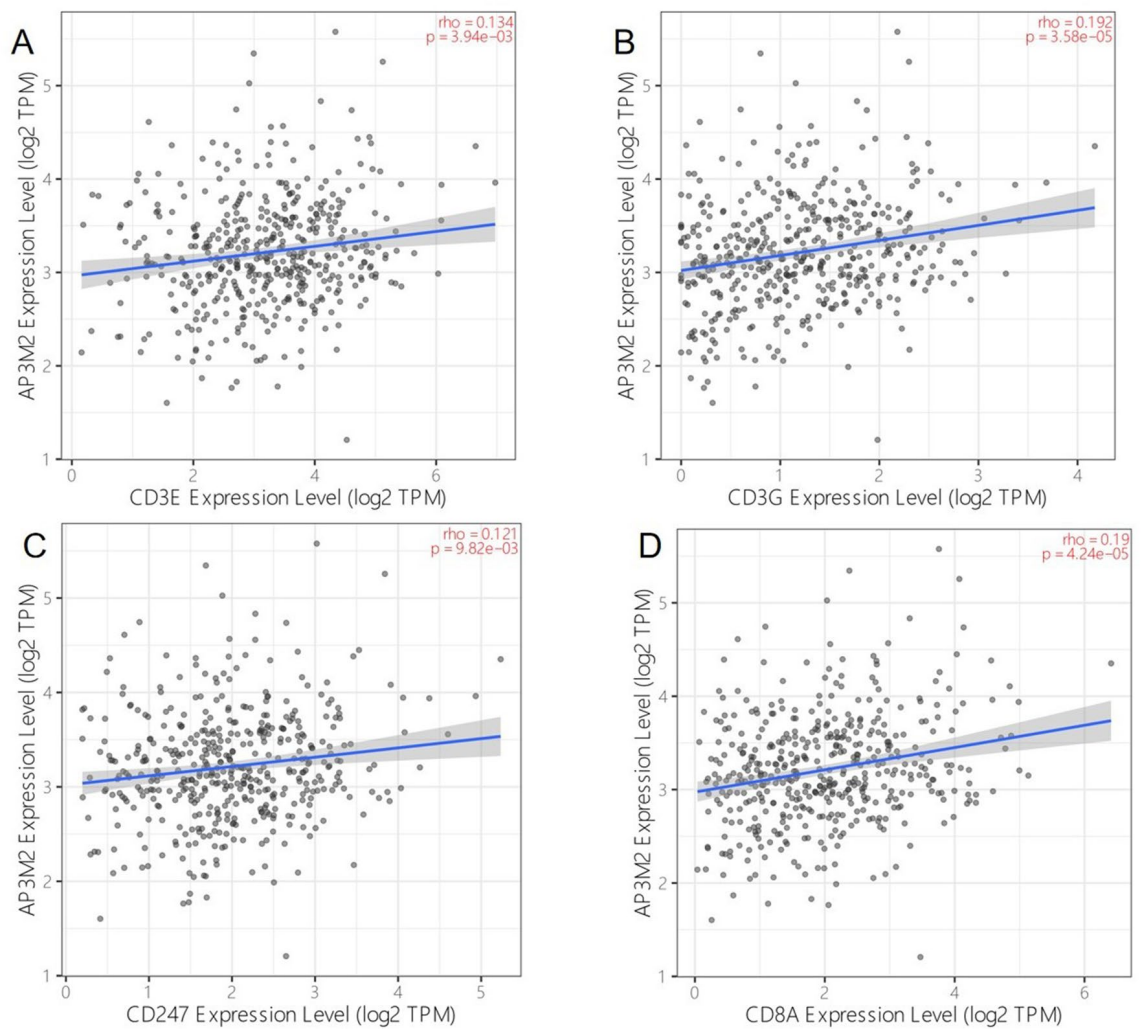
The relationship between AP3M2 expression and genes enriched in T-immune: (**a**) The relationship between AP3M2 expression and CD3E. (**b**) The relationship between AP3M2 expression and CD3G. (**c**) The relationship between AP3M2 expression and CD247. (**d**) The relationship between AP3M2 expression and CD8A.

### Transcription factors of AP3M2

Through LASAGNA database, we found 86TFs associated with AP3M2, and UCSC database has 4 TFs, the intersection of the TFs are WT1 (*p*-value = 5.00E−05), CTF (*p*-value = 6.75E−04), TBP (*p*-value = 5.00E−05), NF-Y (*p*-value = 5.00E−05).

### AP3M2 in tumor-infiltrating immune cells in CRAC

**Figure 7:**
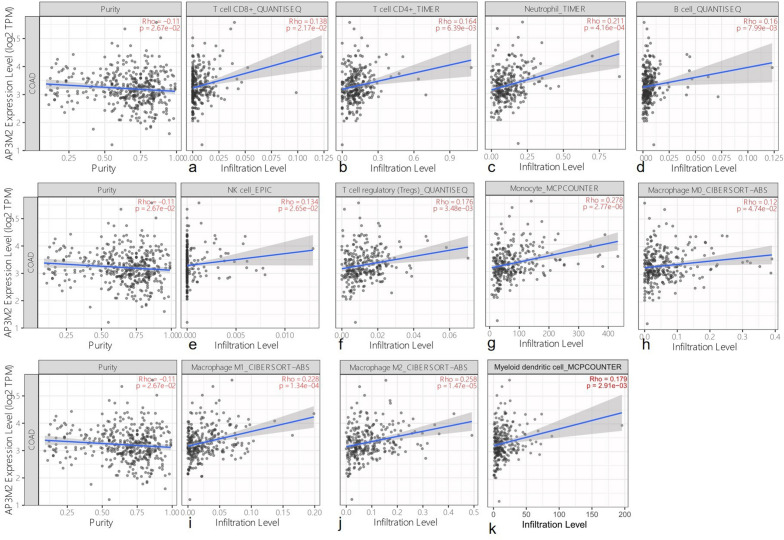
AP3M2 in tumor-infiltrating immune cells in colon cancer: (**a**) The relationship between AP3M2 and CD4+ T cells. (**b**) the relationship between AP3M2 and CD8+ T cells. (**c**) the relationship between AP3M2 and Neutrophil cell. (**d**) the relationship between AP3M2 and B cells. (**e**) the relationship between AP3M2 and NK cells. (**f**) The relationship between AP3M2 and Tregs cells. (**g**) The relationship between AP3M2 and Monocyte cells. (**h**) The relationship between AP3M2 and macrophage M0 cells. (**i**) The relationship between AP3M2 and macrophage M1 cells. (**j**) The relationship between AP3M2 and macrophage M2 cells. (**k**) The relationship between AP3M2 and myeloid-derived suppressor cells.

In order to reveal the relationship between AP3M2 and immune regulation in colon cancer, we then explore the gene and immune cell infiltration through TIMER database. AP3M2 was positively associated with CD8+ T cells (r = 0.14), CD4+ T cells (r = 0.16), Neutrophil cells (r = 0.22), B cells (r = 0.16), NK cells (r = 0.13), Tregs cells (r = 0.18), Monocyte cells(r = 0.28), macrophage M0 cells(r = 0.12), macrophage M1 cells (r = 0.23), macrophage M2 cells(r = 0.26), myeloid-derived suppressor cells(r = 0.16), but negatively associated with B cell memory (r = − 0.15), B cell plasma(r = − 0.13) (Fig. 7).

**Figure 8:**
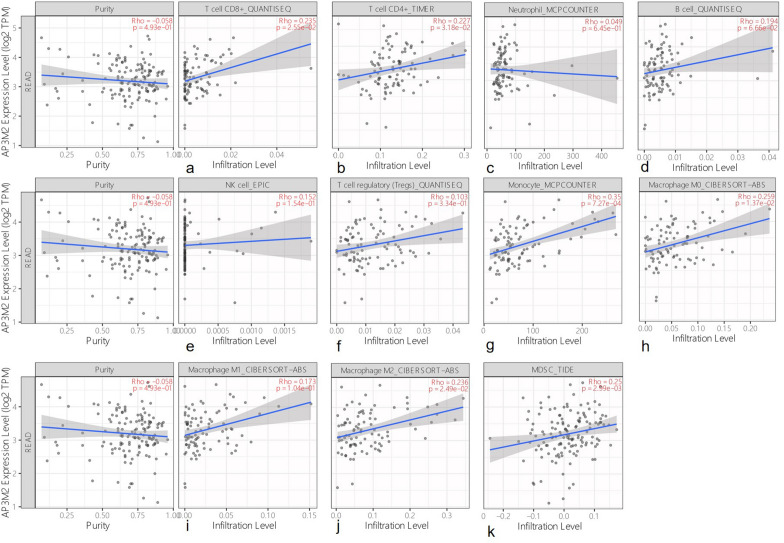
AP3M2 in tumor-infiltrating immune cells in rectal cancer: (**a**) the relationship between AP3M2 and CD4+ T cells. (**b**) The relationship between AP3M2 and CD8+ T cells. (**c**) The relationship between AP3M2 and Neutrophil cells. (**d**) The relationship between AP3M2 and B cells. (**e**) The relationship between AP3M2 and NK cell. (**f**) The relationship between AP3M2 and Tregs cells. (**g**) The relationship between AP3M2 and Monocyte cell. (**h**) The relationship between AP3M2 and macrophage M0 cells. (i) the relationship between AP3M2 and macrophage M1 cells. (**j**) The relationship between AP3M2 and macrophage M2 cells. (**k**) The relationship between AP3M2 and myeloid-derived suppressor cells.

We also explore the gene and immune cell infiltration in rectal cancer through TIMER database. AP3M2 was positively associated with CD8+ T cells (r = 0.24), CD4+ T cells (r = 0.23), Monocyte cells (r = 0.35), macrophage M0 cells (r = 0.26), macrophage M2 cells (r = 0.24), myeloid-derived suppressor cells(r = 0.25), but negatively associated with B cell memory (r = − 0.22), B cell plasma(r = − 0.23) and not related with NK cell, Neutrophil cell, Tregs cell, macrophage M1 cell, B cell. (Fig. 8).

### The relationship of AP3M2 and other genes

**Figure 9:**
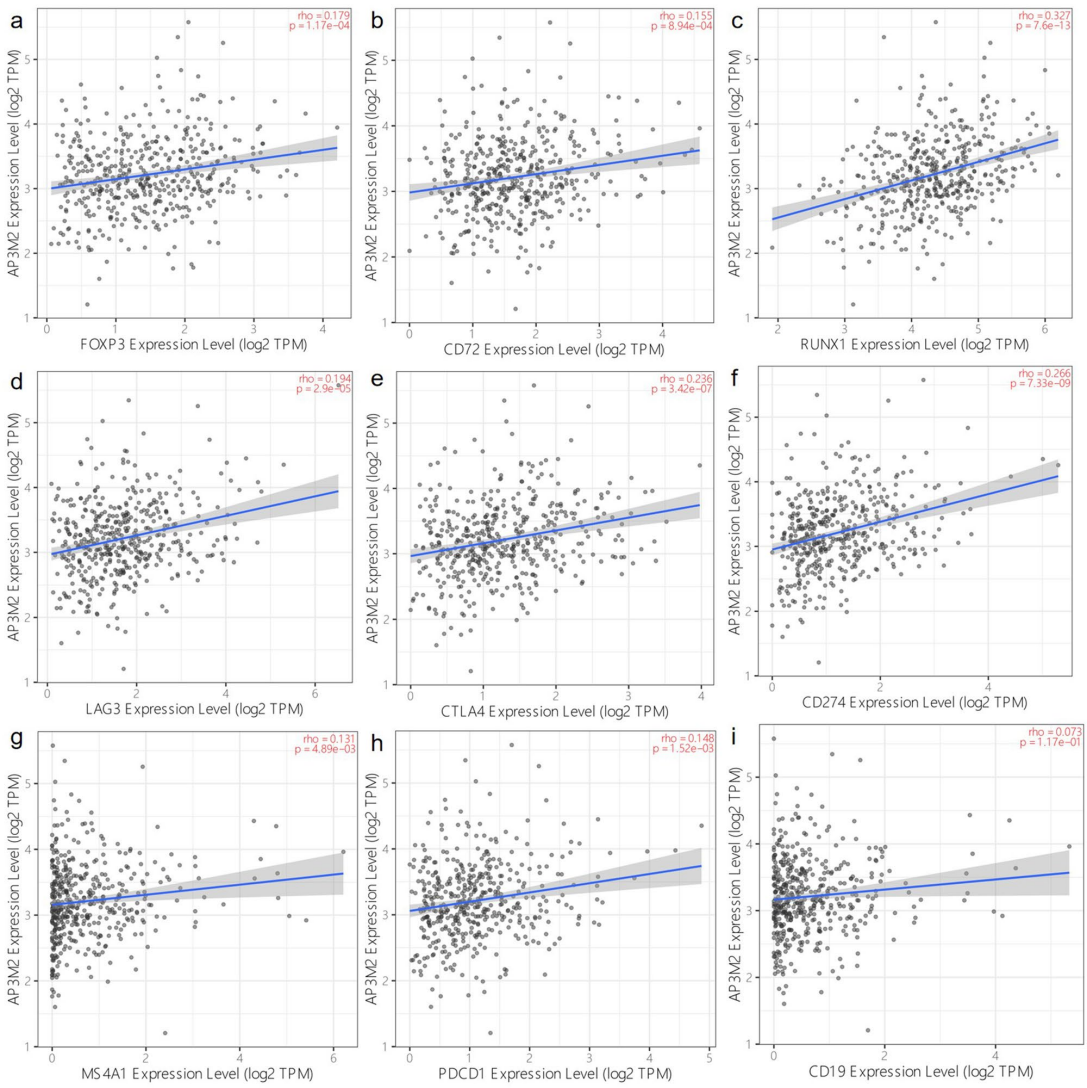
Relationship of AP3M2 expression and other genes in colon cancer: (**a**) The relationship of AP3M2 expression and FOXP3. (**b**) The relationship of AP3M2 expression and CD72. (**c**) The relationship of AP3M2 expression and RUNX1. (**d**) The relationship of AP3M2 expression and LAG3. (**e**) The relationship of AP3M2 expression and CTLA4. (**f**) The relationship of AP3M2 expression and PD-L1. (**g**) The relationship of AP3M2 expression and CD20. (**h**) The relationship of AP3M2 expression and PD1. (**i**) The relationship of AP3M2 expression and CD19.

**Figure 10:**
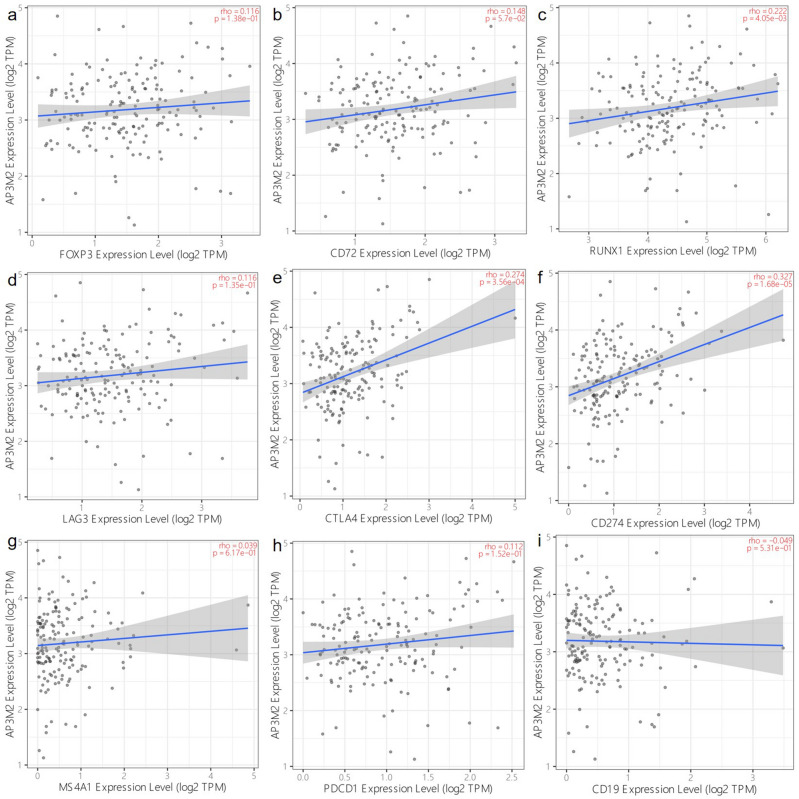
Relationship of AP3M2 expression and other genes in rectal cancer: (**a**) The relationship of AP3M2 expression and FOXP3. (**b**) The relationship of AP3M2 expression and CD72. (**c**) The relationship of AP3M2 expression and RUNX1. (**d**) The relationship of AP3M2 expression and LAG3. (**e**) The relationship of AP3M2 expression and CTLA4. (**f**) The relationship of AP3M2 expression and PD-L1. (**g**) The relationship of AP3M2 expression and CD20. (**h**) The relationship of AP3M2 expression and PD1. (**i**) The relationship of AP3M2 expression and CD19.

Then we investigated genes associated with immune response through TIMER database, we found AP3M2 was positively related with FOXP3(r = 0.18), CD72(r = 0.16), RUNX1(r = 0.33), LAG3(r = 0.19), CTLA4(r = 0.24) PD-L1(CD274) (r = 0.27), CD20(MS4A1) (r = 0.13), PD1(CD279, PDCD1) (r = 0.15) and irrelated with CD19 in colon cancer. However, AP3M2 have no relationship with these genes except CD274(r = 0.33), RUNX1(r = 0.22), CTLA4(r = 0.27) in rectal cancer. (Figs. 9, 10).

**Figure 11:**
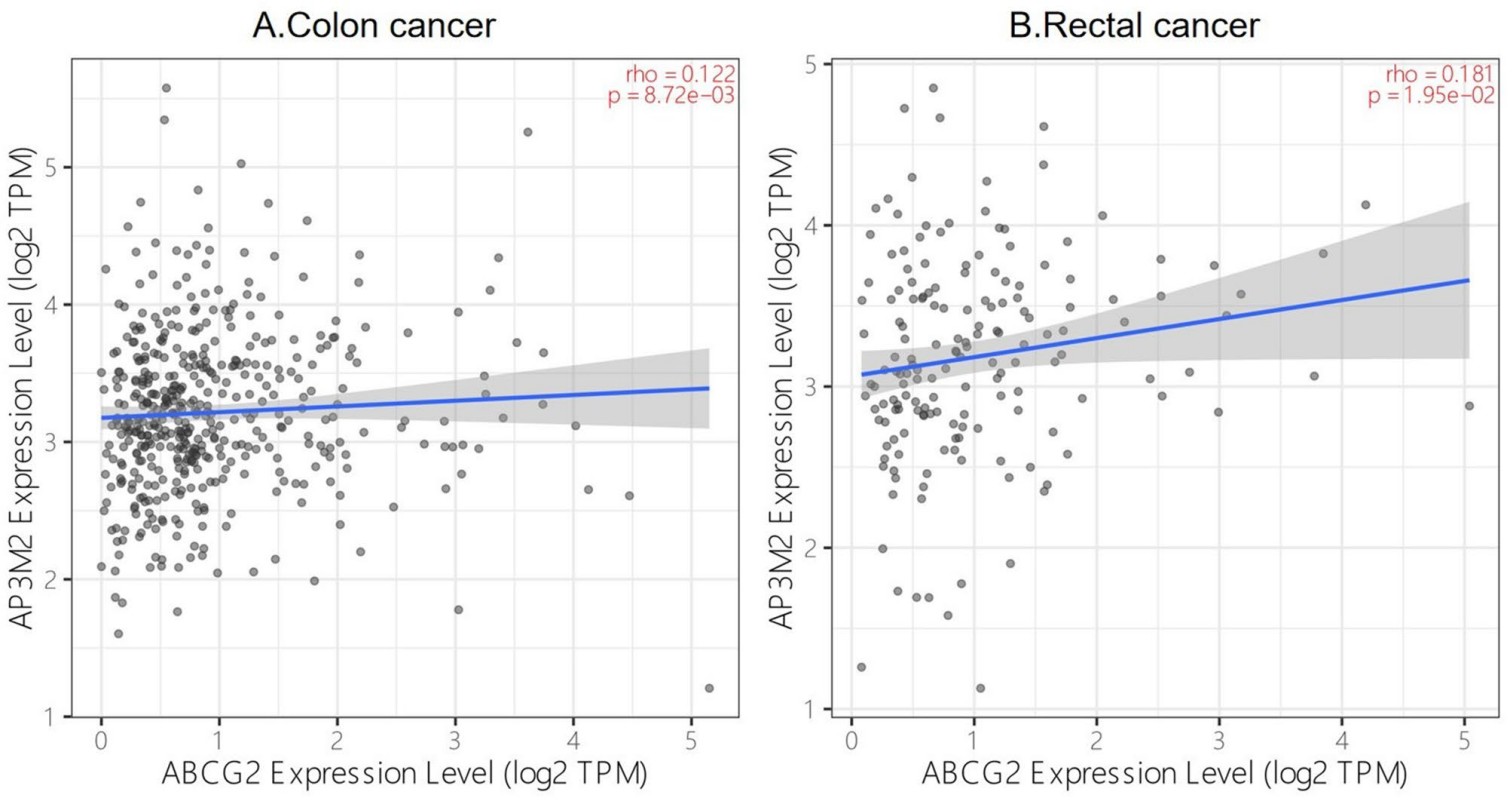
Relationship of AP3M2 expression and ABCG2 in CRAC: (**a**) the relationship between AP3M2 and ABCG2 in colon cancer. (**b**) The relationship between AP3M2 and ABCG2 in rectal cancer.

Moreover, we investigated the relationship between AP3M2 and ABCG2, which was confirmed the energetic association with oxaliplatin resistance. The relationship between AP3M2 and ABCG2 was positive in colon cancer (r = 0.12) and rectal cancer (r = 0.18). (Fig. 11).

### The expression of AP3M2 in colon cancer samples and adjacent normal tissues.

**Figure 12:**
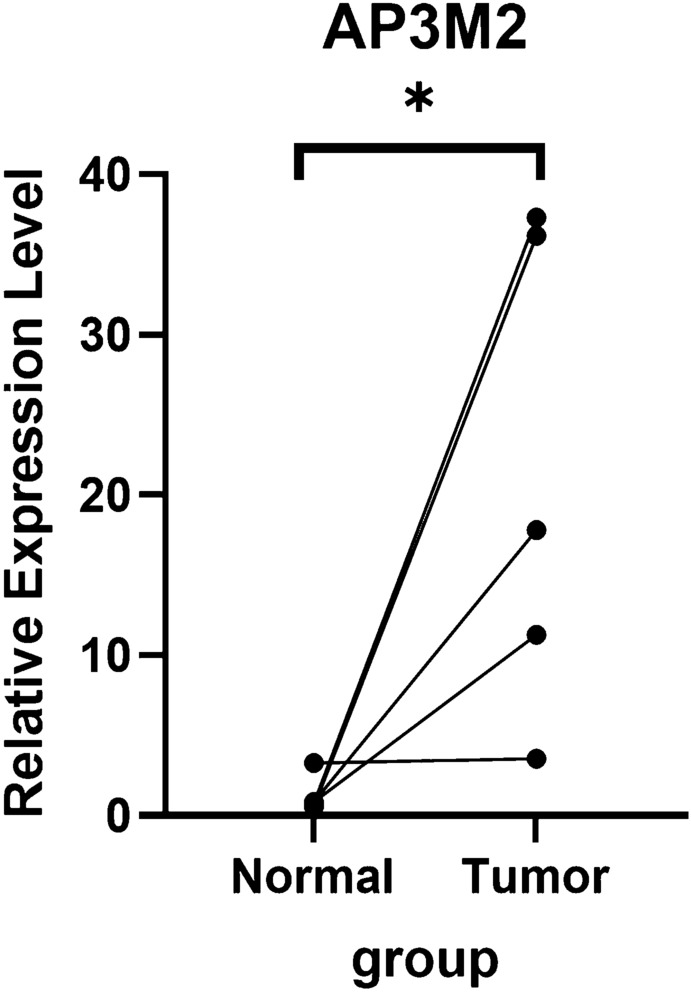
The expression of AP3M2 in colon cancer samples and adjacent normal tissues.

Real-time PCR was used to assess the expression of AP3M2 in colon cancer

samples and adjacent normal tissues. The level of AP3M2 expression was higher in the tumor group compared to the normal group (*p* < 0.05) (Fig. 12).

## Discussion

Diseases associated with AP3M2 include Farber Lipogranulomatosis and Generalized Epilepsy With Febrile Seizures Plus^10^. Several studies showed that AP3M2 lends credence to a functional role in alcohol preference and withdrawal through GABAergic transmission^4,11,12^. Few studies reveal its role in in tumorigenesis. AP3M2 plays different roles in various cancers, it is an oncogene in Breast invasive carcinoma, Cholangiocarcinoma, Stomach adenocarcinoma, Colon adenocarcinoma, Rectum adenocarcinoma, Esophageal carcinoma, Head and Neck squamous cell carcinoma, Liver hepatocellular carcinoma and Lung squamous cell carcinoma. Meanwhile, it plays an antioncogene role in Glioblastoma multiforme, Kidney Chromophobe and Thyroid carcinoma^13,14^.In our study, enhanced expression of AP3M2 predicts poor prognosis in patients with colon adenocarcinoma, but not rectal adenocarcinoma. Furthermore, AP3M2 might not be some clinic factor such as different stages, age groups, or gender of colorectal cancer. as well as prognostic in rectal cancer. But it is a prognostic biomarker for colon cancer. At the same time, AP3M2 seems can be a biomarker between colon cancer and colonic adenomas, liver metastasis, lung metastasis, colonic polyp. Available databases such as TCGA, GEO do not provide data on gene expression between rectal cancer and other rectal diseases,which prevents “validation” of our findings with clinical samples.

In the last 15 years, a significant progress in the management of CRC was achieved with several new agents licensed (irinotecan, oxaliplatin, capecitabine, bevacizumab, cetuximab and panitumumab)^15,16^. The chemotherapy agents of choice for adjuvant treatment of colorectal cancer with stage higher than T3N0M0 after resection are 5-fluorouracil (5-FU) and oxaliplatin. In the case of metastatic colorectal cancer (mCRC), oxaliplatin and irinotecan are considered as the most significant cytotoxic compounds^17–19^.

Resistance to oxaliplatin chemotherapy has become a significant cause for treatment failure and CRC-related death^20^. In our study, AP3M2 was found to be associated with oxaliplatin response in colon cancer. Moreover, we observed an association between AP3M2 and the NF-κB Signaling Pathway with a positive regulation. Studies have revealed NF-κB as one of the stem cell regulatory pathways frequently dysregulated in tumor cells, participating in the regulation of inflammation and leading to chemoresistance against anti-cancer drugs. One of the crucial factors in oxaliplatin resistance is NF-κB via autocrine signaling through IL-1 and IL-8^21–23^. These findings suggest that AP3M2 could indicate oxaliplatin resistance through the NF-κB Signaling Pathway. Besides, overexpression of ABCG2 in oxaliplatin-resistant cells aims to escape from apoptosis induced by Hsi-Hsien Hsu^24^. AP3M2 expression is positively associated with ABCG2 in our study, we hypothesized that AP3M2 might affect the expression of ABCG2 in some way, influencing the acquired drug resistance of Oxaliplatin. However, oxaliplatin seems to induce immunogenic signals on the surface of cancer cells before apoptosis, triggering interferon-gamma production and interaction with toll-like receptor 4 on dendritic cells, resulting in the immunogenic death of cancer cells^25^. This contradicts our conclusion that the overexpression of AP3M2 is positively associated with dendritic cell infiltration in colon adenocarcinoma.

Tumor-infiltrating immune cells (TICs) in the context of tumor microenvironment (TME) are closely associated with clinical outcomes and can regulate tumor development in some way^26^ The promotion of tumor cell proliferation, growth and survival could be triggered by cytokines produced by immune cells^27^. Previous studies revealed that M2 macrophages exhibited pro-tumoral effects through interacting with with various cells, including T helper 2 cells, cancer-associated fibroblasts, cancer cells, regulatory T cells (Tregs), plasmacytoid dendritic cells, and myeloid-derived suppressor cells^28^. This further explains that AP3M2 is positively correlated with M2 macrophage levels and myeloid-derived suppressor cells in CRAC in our study. On the other hand, our studies revealed that AP3M2 is related with response to hypoxia, Tumor-tissue hypoxia can convert M1 macrophages to M2 macrophages^29^. Moreover, AP3M2 overexpression in colorectal adenocarcinoma can also increase the amount of immunosuppressive cells, such as regulatory T cells (Treg) and medullary inhibitory cells, to evade immune surveillance, the numbers of these immunosuppressive cells are closely related to a poor prognosis^30,31^. Recent advances in the field^32^ of tumor immunology reveals that Tregs are believed to promote tumor development by inefficient activation of the immune system against tumor cells. FOXP3^33,34^, CD72^35,36^, RUNX1^37^, LAG3^38^, CTLA4^39,40^ are related to Tregs in the presence of effector cells and contribute to their suppressive activity, suggesting that depletion of Tregs is believed to be a valid therapeutic approach in many cancer types. In our study, FOXP3(r = 0.179), CD72(r = 0.155), RUNX1(r = 0.327), LAG3(r = 0.194), CTLA4(r = 0.236) have a positive correlation with AP3M2 in Colon Neoplasm, but no irrelevancy in rectal tumor. Immune check point inhibitors with early Treg depletion might represent an effective strategy for the therapy of Colon Neoplasm^32^, and AP3M2 might be worth considering. This explains the different prognosis of AP3M2 in colon and rectal cancer.

The quantity of the immune infiltrate is crucial for mounting an efficient antitumor response. In this regard, the transcriptional level of key genes involved in T cell, and B cell function may reveal deregulation^41^.We identified hub genes (CD3E, CD3G, CD247, CD8A, CD19, CD20(MS4A1),CTLA4,CD279) that were possibly synclastic related to AP3M2 and contribute to the immune response in Colonic Neoplasm. Studies have shown that the expression of CD3G, CD8A, CD19, MS4A1 is related to immune system activation and significantly associated with prognostic factors or disease survival^42–46^. These genes play a possible role in immune-mediated pathways to achieve the elimination of neoplastic cells, affecting the prognosis^47^. On the contrary, CTLA4 (Cytotoxic T lymphocyte antigen 4, CD152), PD1 (programmed cell death 1, CD279) receptors on the surface of activated T cells inhibit the activation of T cells by interacting with their ligands^48–51^. CD4+ memory activated and resting T cells and Tregs were negatively correlated with CD3E^41^. This supports the idea that AP3M2 affects prognosis through influencing the immune response. More notably, AP3M2 is independent of Immune Regulation in rectal cancer, which explains the different prognosis manifestation in the rectum instead.

Programmed death-ligand 1 (PD-L1, also known as B7-H1 and CD274) is one of the most critical immune checkpoints in CRAC. The binding of programmed cell death protein 1 (PD-1) and its ligand PD-L1 plays a negative regulatory role in the activation of T cells^52,53^. Previous studies have demonstrated that PD-L1 is overexpressed in various cancers including CRC^54,55^. PD-L1 expression is associated with a poor prognosis and inhibits cell proliferation^56^, migration, and invasion in CRC^57^. Besides, PDL1 may regulate immune-independent and intrinsic functions of tumor cells that include tumor cell apoptosis and autophagy^58,59^. Simultaneously, according to the current literature, the expression of PD-L1 is regulated by hypoxia-inducible factor-1 (HIF-1), which could bind to hypoxic response element (HRE) and upregulate the PD-L1 expression, simultaneously cause T-cell apoptosis and function inhibition^60,61^. NF-kB was also important transcription factors upregulating PD-L1^62–64^. NK cell infiltration induces the expression of immune checkpoints such as PD‐1 with upregulated Th1 lymphocyte and cytotoxic T cell^65^. In our study, AP3M2 was found to be positively related to PD-L1(CD274), hypoxic response, NF-kB pathway, and NK cell infiltration in colorectal adenocarcinoma. Therefore, we speculate that AP3M2 plays a possible role in colorectal cancer immunotherapy.

Our study comprehensively analyzed the role of AP3M2 in colorectal adenocarcinoma, incorporating data from both TCGA and GEO. Nevertheless, our study has inherent limitations: first, all analyses were based on RNA sequence data. Second, population heterogeneity might exist across different datasets in this study. The absence of in vivo and in vitro experiments to validate our findings should be noted. Further research is warranted to investigate how AP3M2 affects drug effectiveness and colon cancer prognosis.

We hypothesize that AP3M2 functions as an oncogene in CRAC. AP3M2 could potentially predict chemotherapy effectiveness and prognosis for colon cancer patients. Moreover, AP3M2 might inhibit tumor growth by influencing tumor-infiltrating immune cells (TICs) in the context of the tumor microenvironment (TME). It is suggested as a novel potential biotarget for therapy.

## Methods

### Statistical analysis

Significance was defined as a *p*-value < 0.05 for gene expression, survival analysis, and protein–protein network relations.

### Patients and data collection

Difference gene expression and clinical material of CRAC patients were downloaded from the TCGA database. Subsequently, the expression data were utilized to analyze mRNA expression levels in cancer tissue compared to normal tissue. (supplemental Fig. 1).

### GEPIA, timer and ualcan database analysis

The transcription level of AP3M2 gene was identified by ualcan database (http://ualcan.path.uab.edu/index.html)^66^ and Timer database(http://timer.comp-genomics.org/)^13,14,67^ The relationship between AP3M2 expression and survival in CRAC was analyzed by GEPIA database (https://www.gepia.cancer-pku.cn/)^68^. The Promoter Methylation level of AP3M2 was also identified by ualcan database. Furthermore, the relationship between AP3M2 and immune regulation was identified by TIMER database.

### ROC plot database analysis and GEO database

ROCPLOT database (http://www.rocplot.org/user/login)^69^ was used to analyze the predictive values of different drugs and the prognostic value of AP3M2 in CRAC. GEO database was used to analyze The Expression of AP3M2 between Acquired Drug-Resistant and Parental Cell Lines.

### David database analysis

KEGG and GO enrichment analysis about AP3M2 was analyzed by David database (https://david.ncifcrf.gov/)^70,71^.

### Other online database analysis

Transcription Factors of AP3M2 was found by LASAGNA database (https://biogrid-lasagna.engr.uconn.edu/)^72^, USSC database (http://genome.ucsc.edu/)^73^,miRDB database(http://mirdb.org/)^74^, targetscan database (http://www.targetscan.org/)^75,76^ and miRWalk database (http://mirwalk.umm.uni-heidelberg.de/) Other genes that might be associated with AP3M2 were explored using Cytoscape^77^based on available public databases.

### Clinical specimen collection and ethics approval

Colon cancer samples and normal tissues were acquired from the Seventh Affiliated Hospital of Sun Yat-sen University. Ethical approval for this study was obtained from the Sun Yat-sen University Health Science Institution Review Board (No. KY-2022–051-02). Samples were collected for real-time PCR.

### Ethics statement

Research have been performed in accordance with the Declaration of Helsinki. All methods were performed in accordance with the relevant guidelines and regulations. All patients provided their informed consent in writing preoperatively.

### Real-time PCR analysis

Total RNA was extracted from colon cancer and adjacent normal tissues using AG RNAex Pro RNA reagent (Accurate Biology, CAT#AG21102) according to the manufacturer's instructions. cDNA was synthesized using the Evo M-MLV reverse transcription Master Mix (Accurate Biology, CAT# AG11706). RT-qPCR was performed with a SYBR Green Pro Tag HS premixed qPCR kit (Accurate Biology, CAT# AG11701). The relative expression of total RNA was calculated using the 2^-ΔΔCt^ method. The primer sequences for all the RNAs used for qPCR are recorded in Supplementary Table 1.

### Supplementary Information

Supplementary Information.

## Data Availability

The datasets presented in this study can be found in online repositories. The names of the repository/repositories and accession number(s) can be found in the article/Supplementary Material.
