# Supplementary material for: Prognostic and immunological role of adaptor related protein complex 3 subunit mu2 in colon cancer
Source: Sci Rep. 2024 Jan 4;14:483. doi: 10.1038/s41598-023-50452-2 (PMC10767120; doi:10.1038/s41598-023-50452-2)
Supplement: Supplementary file 1 — Supplementary Information. [file 41598_2023_50452_MOESM1_ESM.docx]

Supplemental figure 1: The mRNA expression levels in colorectal cancer tissue compared to the normal.


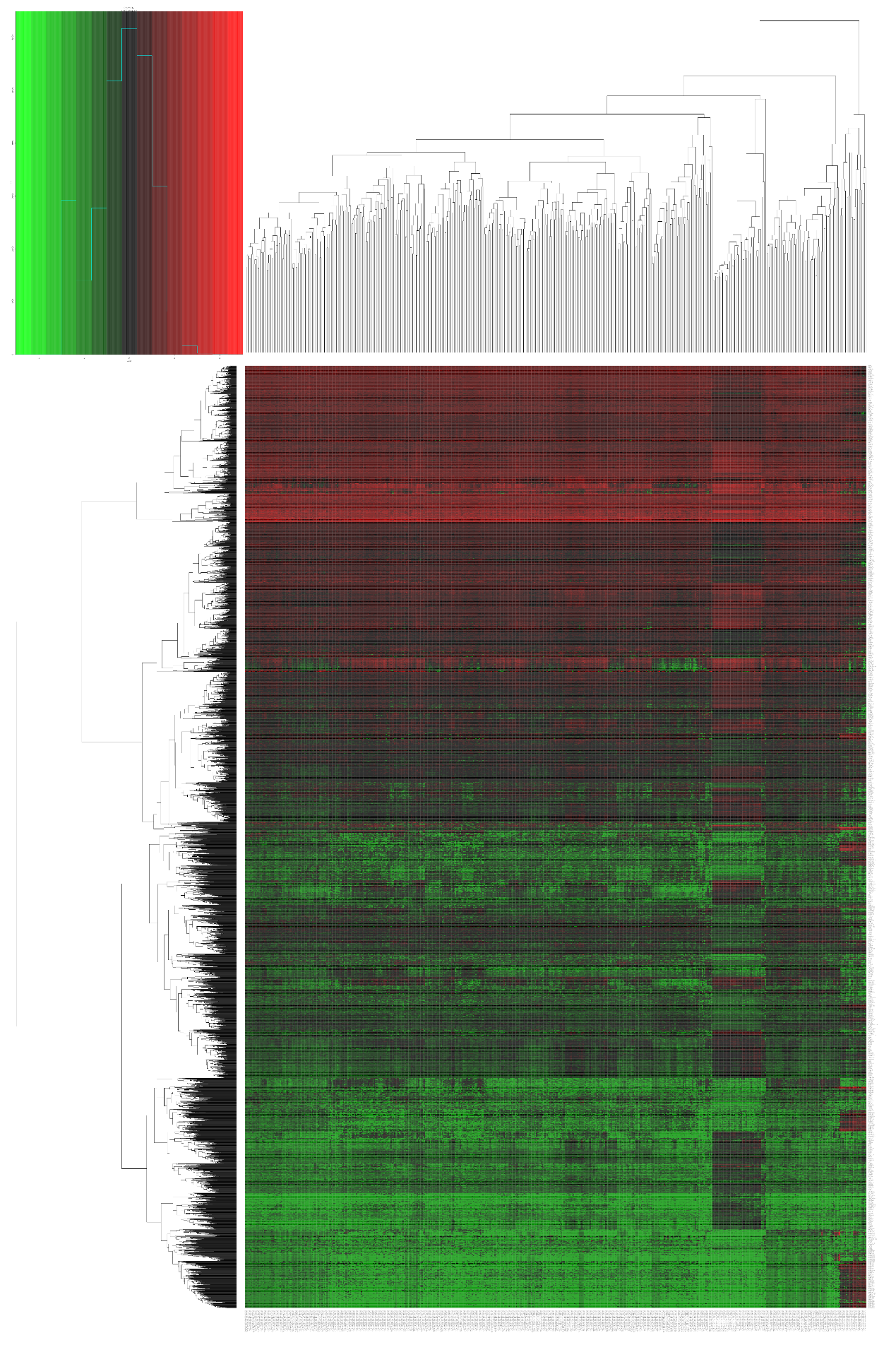


Supplemental figure 2: The relationship between AP3M2 expression and Clinical features:


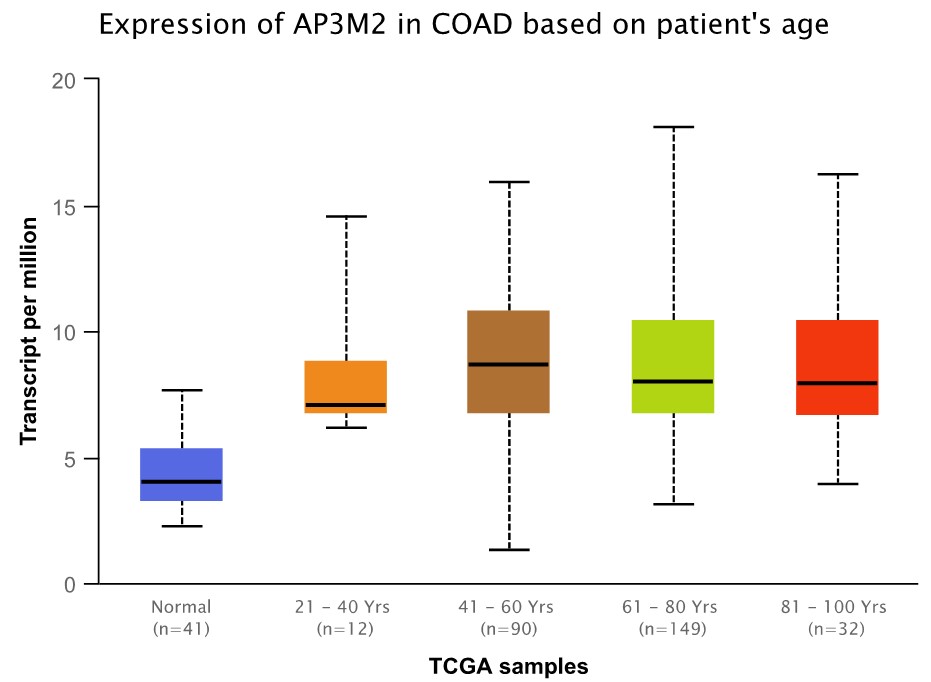


(a)The box plot comparing specific AP3M2 expression in different ages was derived from the ualcan database in colon cancer.
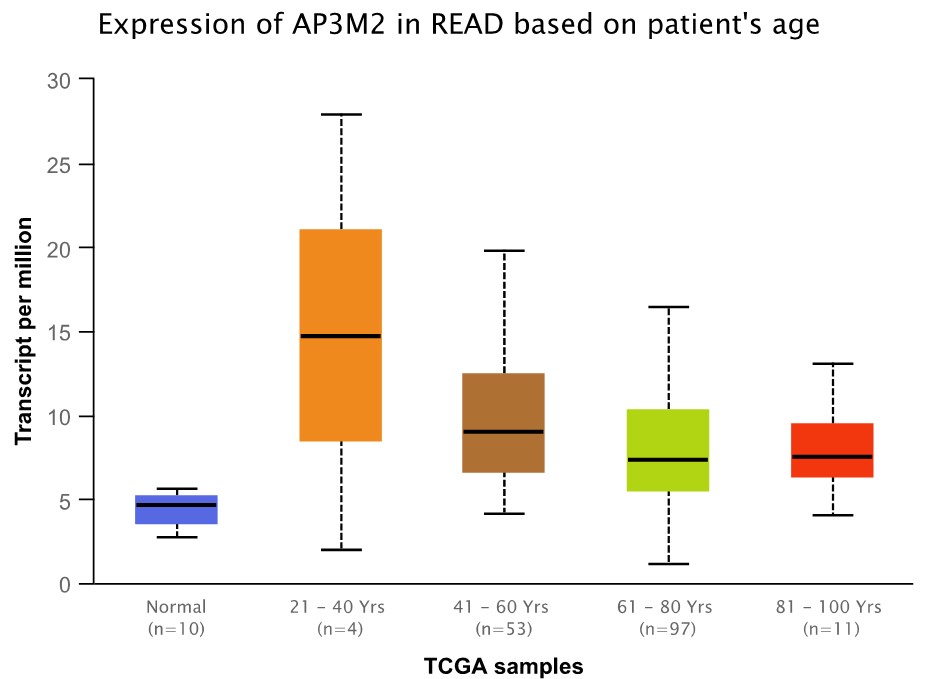


(b) The box plot comparing specific AP3M2 expression in different ages was derived from ualcan database in rectal cancer.
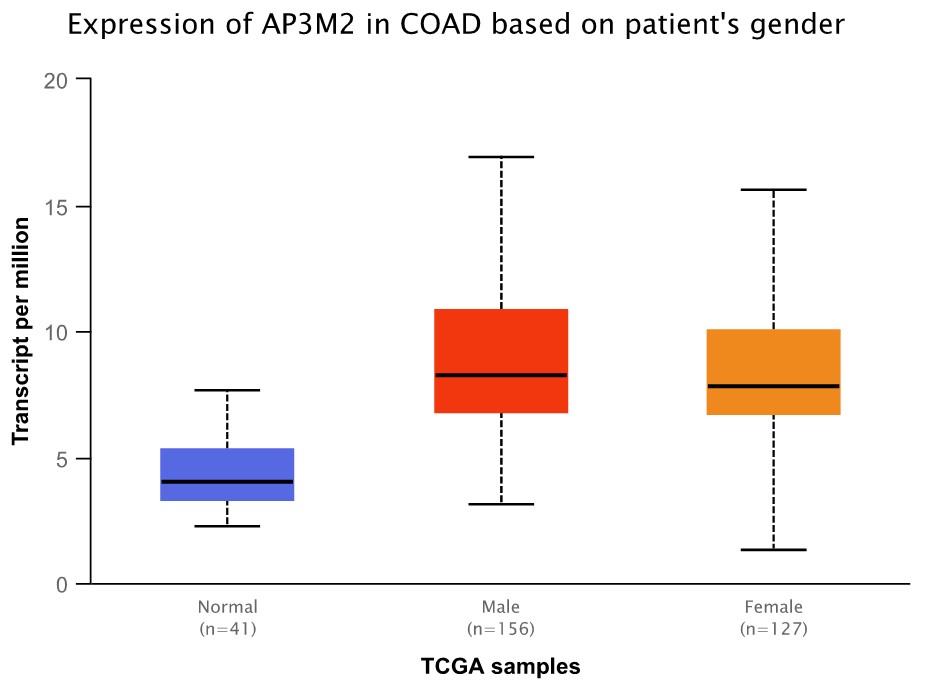


(c)The box plot comparing specific AP3M2 expression in different genders was derived from ualcan database in colon cancer.
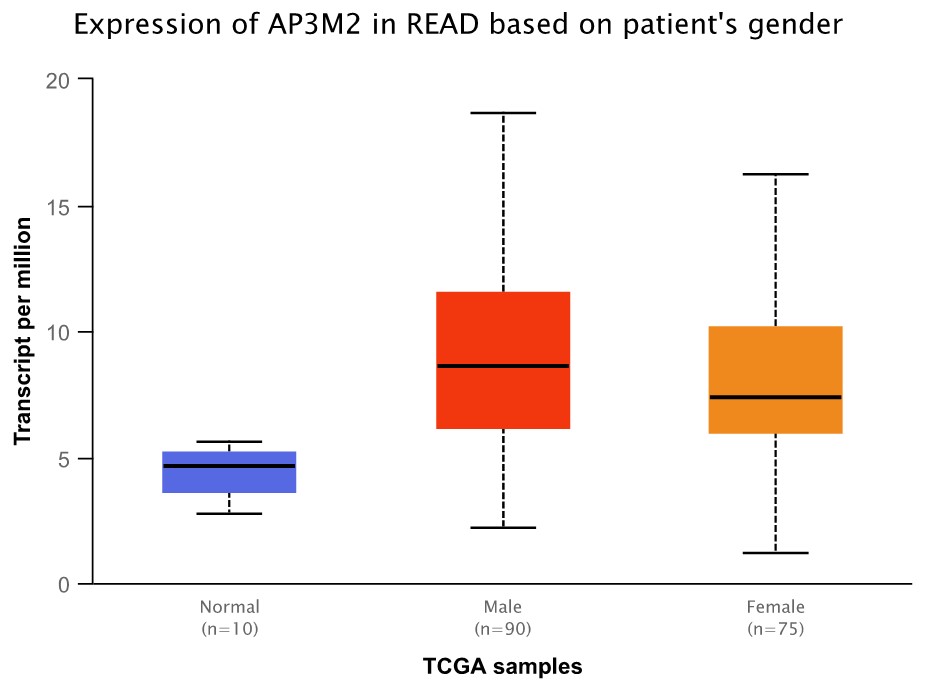


(d)The box plot comparing specific AP3M2 expression in different gender was derived from ualcan database in rectal cancer.

[Supplementary Table 1](https://www.frontiersin.org/articles/10.3389/fmolb.2023.1163977/full#SM1). Primer sequences of mRNA for real-time polymerase chain reaction.

| Target | Sequence of primers |
| --- | --- |
| GAPDH-Forward | GGAGCGAGATCCCTCCAAAAT |
| GAPDH-Reward | GGCTGTTGTCATACTTCTCATGG |
| AP3M2-Forward | TTGAGGCGCAAGAGAGAGCTA |
| AP3M2-Reverse | GTCCACCACTCGGTGAAGAAA |
